# Death Rate

**Published:** 1870-07

**Authors:** 


### DEATH RATE.

THAT is a very healthy place where only twelve persons die of disease, in a year, out of a population of a thousand souls.

A few years ago the death rate of New York City was thirty-five in a thousand. In the last two or three years it has averaged thirty.

In Philadelphia there is a possibility of living a hundred and fourteen years, but the average life is thirty-five years.

Evenness of temper, cleanliness and temperance, are the characteristics of Quakers, as a community, everywhere, and these virtues give them an average of life twelve years longer than that of their neighbors, and between twenty and sixty years, of all. Fewer Quakers die, by twenty per cent., than others.

